# Reinforcement of Epoxy Composites with Graphite-Graphene Structures

**DOI:** 10.1038/s41598-019-52751-z

**Published:** 2019-11-07

**Authors:** A. S. Mostovoy, A. V. Yakovlev

**Affiliations:** Yuri Gagarin State Technical University of Saratov, Polytechnichskaya St., 77, 410054 Saratov, Russia

**Keywords:** Chemical engineering, Graphene, Composites, Mechanical properties

## Abstract

As a result of the research, the possibility of directional control of the operational properties of epoxy composites by the use of small additives of thermally expanded graphite-graphene structures has been proved. The rational content of the structuring additive in the composition of the epoxy composite (0.05 parts by mass.) was selected, which ensured an increase in the studied complex of physico-mechanical properties. The influence of thermally expanded graphite on the process of structure formation of an epoxy composite has been established. The addition of thermally expanded graphite increases thermal, fire and heat resistance as well as the coefficient of heat-conducting epoxy composite.

## Introduction

Currently, polymer composite materials which combine unique properties, high deformation and strength characteristics, and at the same time low specific weight are widely applied in industry^1–3^. The most common type of a binder in industry is various epoxy oligomers. The most important problem is the fragility of this type of material, so there are various types of system modifications^2,3^. The use of plasticizers and fillers provides necessary performance characteristics of composite materials. The addition of plasticizers provides the elasticity of polymeric materials and changes their glass transition temperature^4^. The addition of fillers allows to increase the mechanical and physicochemical characteristics of composites^1–7^. To provide polymer composite materials and their products with better operational characteristics, various modifying additives and fillers are used^1–5^. Mineral fine-dispersed fillers such as talc, basalt, and chromite are wide spread, however, the expected useful effect often exceeds the actual one observed in practice, which is due to the low adhesion of mineral materials to polymer matrices and their tendency to agglomerate. As nanofillers, various types of carbon nanotubes, fullerenes, graphenes, astralenes, technical carbons, as well as titanium and silicon dioxide, diamond batch, white carbon black, etc., are widely used^8–14^. Due to the high specific surface, thermal stability and excellent mechanical characteristics, carbon materials (graphite and graphene oxides, nanotubes, nanofibres) are promising for widespread use in lightweight polymer composites, providing them with enhanced performance characteristics^14–21^. Despite the enormous number of works devoted to the study of the effects of various modifying systems (including carbon), there are still insufficiently studied issues related to the influence of modifiers on the structure formation processes, the structure and performance characteristics of polymer composite materials, which predetermines the direction of the research of this work.

The main goal of this research is to enhance physicochemical and mechanical properties of epoxy composites using thermally expanded graphite obtained by thermal exfoliation of electrochemically oxidized graphite powder.

## Materials and Research Methods

The compositions were developed on the basis of epoxy resin ED*-*20 (GOST 10587-93) because it has low viscosity, a narrow limit of the epoxy groups content, the stability of physico-chemical properties. As a hardener of epoxy oligomer, an amine type hardener was used - polyethylene polyamine (PEPA) (TS 6-02-594-85), capable of forming a three-dimensional network structure without heating.

For the plasticization of epoxy composites, oligo(resorcinophenyl phosphate) with terminal phenyl groups (ORPP) purity: 99% manufactured by ICL Industrial Products America Inc. (USA), was used in the work. Its chemical formula is shown in Supplementary Fig. S1.

ORPP – oligomeric halogen-free plasticizer with flame retardant properties. The choice of ORPP is due to the presence of combustion inhibitor – phosphorus (10,7%). During thermal decomposition of the composite, the presence of phosphorus provides an increase in the yield of carbonized structures, which are the physical barrier for the inter-diffusion of the oxidant and combustible gases to the combustion zone, which, in general, reduces the flammability of epoxy composite^22^.

The ratio of epoxy oligomer, plasticizer and hardener was previously determined experimentally: 100 parts by mass of ED-20, 40 parts by mass of ORPP and 15 parts by mass of PEPA^22^.

Thermally expanded graphite obtained by thermal exfoliation of electrochemically oxidized and hydrolyzed graphite was used as a structuring additive. Electrochemically oxidized graphite (OG) was obtained by electrochemical oxidation of natural dispersed graphite with a particle size of <160 μm in 58% HNO_3_ in the galvanostatic mode at the current I = 100 mA/g of graphite, with the capacity Q = 400 mA h/g^23^. At the next stage, the hydrolysis of the synthesized graphite nitrate was carried out: the reaction mixture was quickly diluted with cold distilled water (t = 15–18 °C) by stirring. Samples were kept in the water for 15 minutes. After dilution, the solid phase was filtered with the help of a Buchner funnel and washed with cold distilled water to pH = 5–7 for wash water. The specific consumption of water for the hydrolysis of graphite nitrate was 100 ml, and for the washing of the obtained oxidized graphite we used 500 ml of H_2_O per 1 g of the product. The drying of hydrolyzed graphite nitrate to constant weight was carried out in the drying oven at 105 ± 2 °C. The particle size of the solid phase in the aqueous suspension is in the range of 10^2^–10^6^ nm, the fraction of exhaust gas with a dimension of 10^2^–10^3^ nm in the suspension is 5–10%. The weighted fraction of the OG is separated from the main OG fraction by decanting. Thermal reduction and exfoliation of the oxidized graphite was carried out under static conditions for 5 seconds at 250 °C in the SNOL – 1.6.2.5.1/9 – I4 muffle furnace. The schematic of overall synthesis strategy of TEG was shown in Supplementary Fig. S2a.

In plasticized epoxy composition TEG was added as a modifying agent (0.01–1.0 parts by weight). To increase the uniformity of distribution and hinder the aggregation of TEG particles, as well as the activation of its surface and binder, ultrasonic treatment of the composition was used. The parameters of the ultrasound exposure: frequency −22 ± 2 kHz, power - 400 W, duration – 60 minutes^4^. The mixture was degassed at 25 ± 5 °C for 30 min under vacuum before curing. The preparation process of TEG/epoxy composites was shown in Supplementary Fig. S2b.

The research was carried out using the following methods:

- determination of bending stress and flexural modulus [ISO 178: 2010],
- determination of strength and modulus of tensile elasticity [ISO 527-2: 2012];
- determination of compressive strength [ISO 604: 2002];
- determination of impact strength [ISO 179-1: 2010];
- determination of Brinell hardness [ISO 2039-1: 2001];
- determination of heat resistance according to Vicat [ISO 306: 2004];
- change in mass, rate of change in mass and magnitude of thermal effects during the heating of the samples was studied using the method of thermogravimetric analysis with the help of a derivator of the “Paulik – Paulik – Erdei” system of the MOM brand Q-1500D under the experimental conditions: weight - 100 mg, medium – air, heating interval - up to 800 °С, heating rate - 10 °С/min, relative error does not exceed 1%;
- the study of the surface morphology of the samples was carried out using a Tescan VEGA 3 SBH scanning electron microscope;
- determination of thermal conductivity and thermal resistance was carried out using the ITP-MG4 “100“instrument [ISO 22007-2: 2015];
- FT-IR spectroscopy of TEG particles was carried out using the Shimadzu IRTracer-100;
- X-ray phase analysis was performed using ARL X’TRA X-ray diffractometer;
- determination of the curing kinetics of the epoxy composition was carried out according to the method described in^24^.

## Experimental Results and Discussion

**Figure 1:**
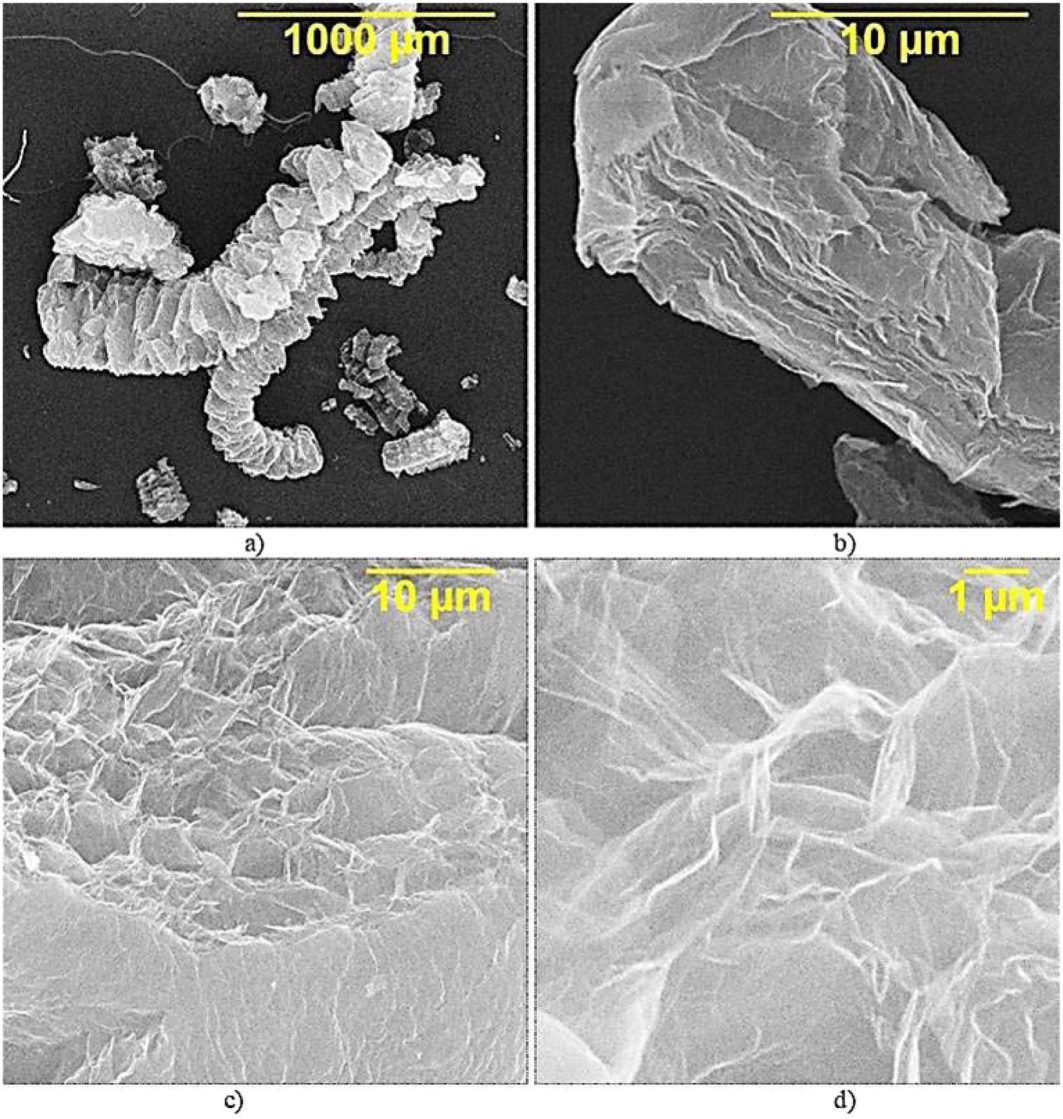
SEM of TEG particles.

According to the data of scanning electron microscopy, during thermal expansion graphite transforms into a worm-like structure with an increased interlayer distance and a highly active, branching, uneven surface, Fig. 1a.

TEG particles have a nano-layered structure, the thickness of the packs of layers is about 100 nm, Fig. 1b, which makes it possible to speak about the formation of particles of a multilayer graphene oxide. The thin leaves of TRG form a complex open cellular microstructure with a pore size of 1–10 microns. In cross section, pores have a polygonal isometric or weakly extended form, Fig. 1c,d.

**Figure 2:**
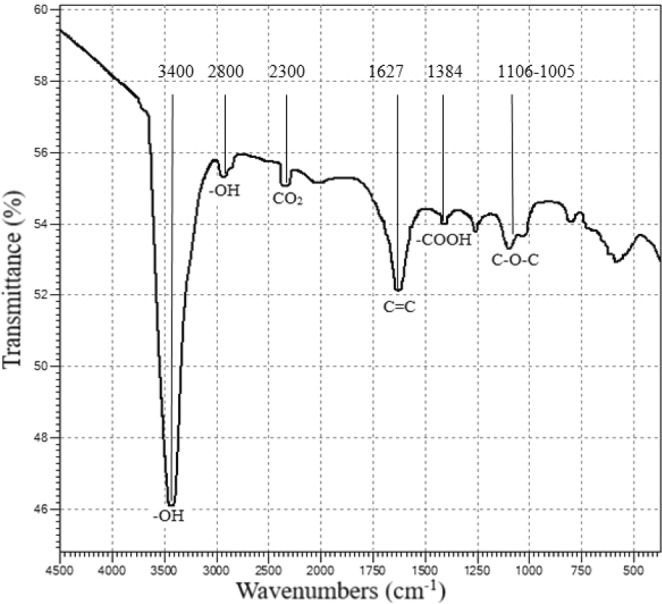
FT-IR spectroscopy of TEG.

A series of signals on the IR-spectra confirm the presence of the form of oxidized graphene. This presence of hydroxyl groups between the graphene layers is the band between 2800 cm^−1^ and 3400 cm^−1^, Fig. 2. The peak at 1627 cm^−1^ is due to the presence of sp^2^-hybridization of C=C in the structure of graphene. The peak at ~2300 cm^−1^ corresponds to the peak of CO_2_ molecules absorbed by the TEG. The band between 1106 cm^−1^ and 1005 cm^−1^ corresponds to C-O-C (epoxy group). The band at 1384 cm^−1^ is the deformation vibration of the carboxyl group.

**Figure 3:**
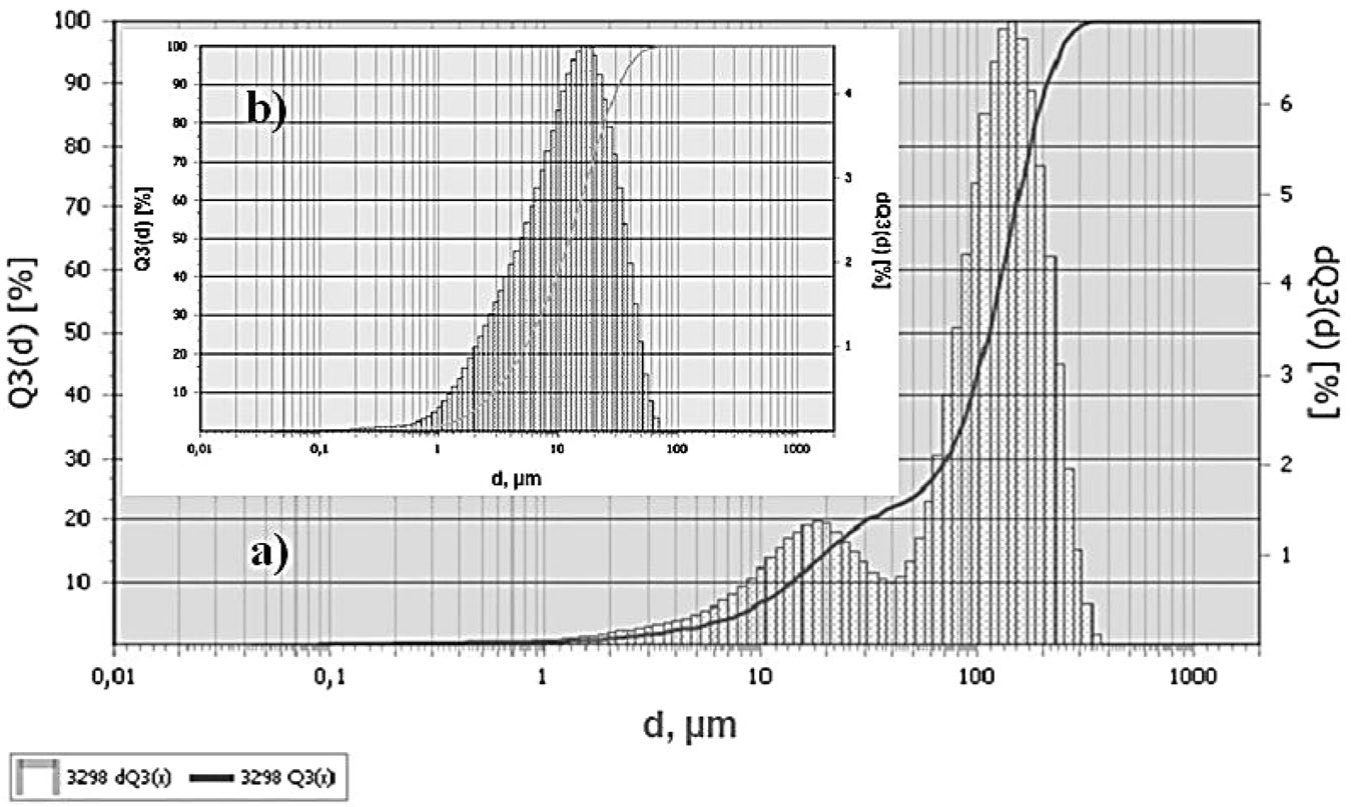
Fractional composition of TEG particles: (**a**) – without ultrasonic dispersion (**b**) – after ultrasonic dispersion.

The fractional composition of TEG is characterized by the bimodal distribution of particles and is represented by particles from 1 to 400 μm, with a predominant number of particles with sizes of 15–20 μm and 140–160 μm, Fig. 3a.

An attempt to use TEG with a particle size of more than 100 µm as a reinforcing filler will lead to the opposite effect – a decrease in the indicators of physico-mechanical properties. Therefore, at the second stage, the method of liquid-phase separation of graphite in the media of the TCPP plasticizer was used to obtain a suspension of graphene particles. Dispersing was carried out using the UZDN-2T ultrasonic disperser with a radiator power of 400 W and a frequency of 22 kHz. Such a combined mechanochemical action led to additional exfoliation and breaking of particles with a large lateral size.

The fractional composition of TEG after ultrasonic dispersion is characterized by a monomodal distribution of particles and is represented by particles from 0.2 to 70 μm, with a predominance of particles with sizes of 10–30 μm, Fig. 3b.

Thus, the analysis of the TEG structure showed that it could be used as a structuring additive for epoxy composites, which should provide an increase in their operational properties.

As a polymer matrix we used previously developed composition consisting of 100 parts by mass of ED-20 epoxy resin, 40 parts by mass of ORPP and 15 parts by mass of a hardener – PEPA. ORPP performs simultaneously the functions of both a plasticizer and a flame retardant. The bending stress doubles and toughness increases by 2 times, and the flammability index - the oxygen index (OI) - increases from 19 to 28% by volume, which enables the material to become flame-retardant^22^.

TEG was added to the epoxy composition in the amount of 0.01–1.0 parts by mass.

**Figure 4:**
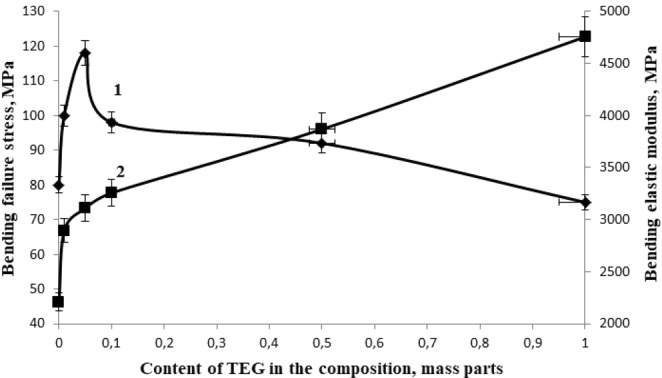
The dependence of the bending failure stress (1) and the bending elastic modulus (2) of the epoxy composite on the TEG content in the composition.

**Figure 5:**
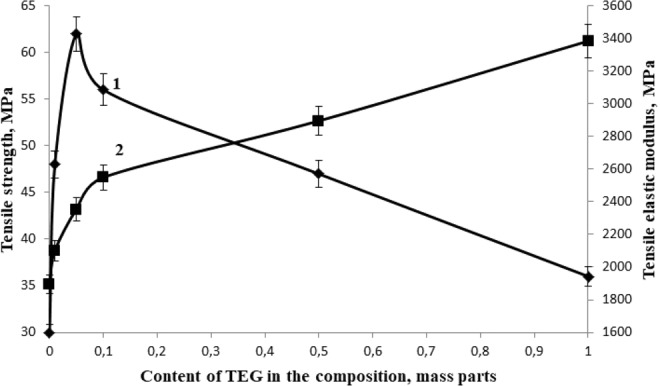
Dependence of tensile strength (1) and tensile elastic modulus (2) of epoxy composite on the TEG content in the composition.

**Figure 6:**
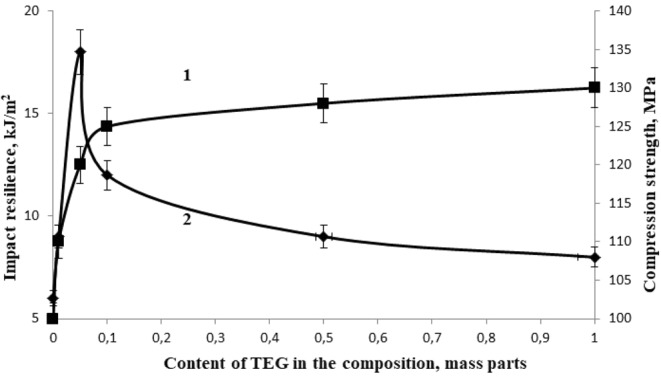
The dependence of compression strength (1) and impact resilience (2) of the epoxy composite on the TEG content in the composition.

The conducted studies have shown that the most rational content of TEG as a structuring additive, providing maximum values of physical and mechanical properties is 0.05 parts by mass, Figs 4–6, at the same time the bending failure stress increases by 48% and the bending elastic modulus increases by 41%, the compression strength increases by 20%, the tensile strength increases by 207% and the tensile elastic modulus increases by 24%, impact resilience increases by 300%.

**Figure 7:**
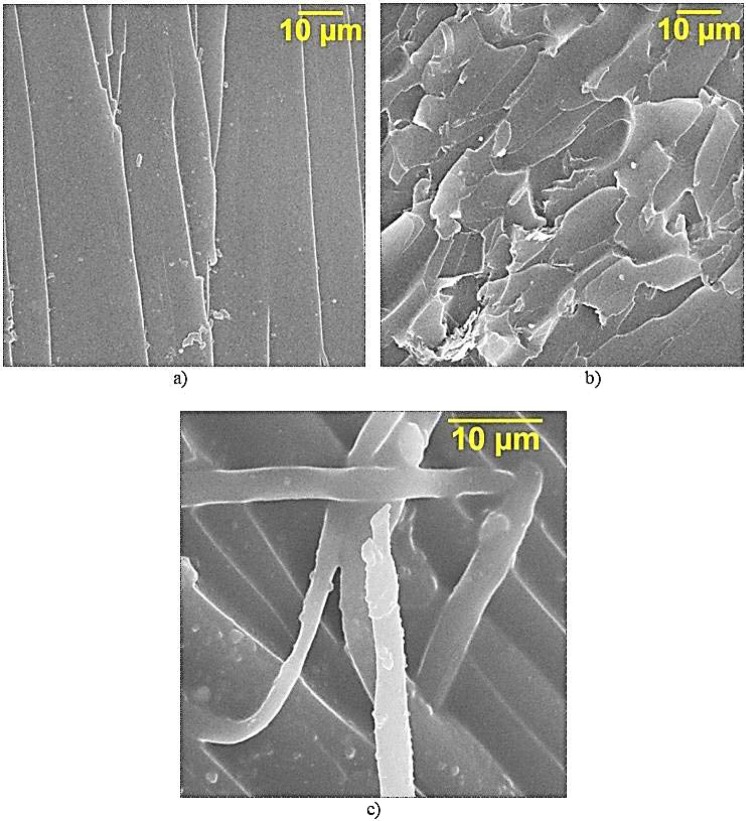
SEM of the surface of the destruction of epoxy composites.

Fractography of the destruction of epoxy composite samples without TEG, Fig. 7a, is characterized by a rather smooth fracture surface, which indicates a low ability to crack resistance. The addition of TEG to the epoxy composition affects the morphology of the matrix — there appear layered structures formed by TEG particles, Fig. 7b. Besides the brittle fracture with the formation of numerous scales, there are local areas in the epoxy composite, indicating the flow of material in the process of its destruction. Moreover, in some places of plastic destruction, pronounced fibrous structures are observed, which are formed as a result of the intensive drawing out of the polymer matrix, Fig. 7c. The increase in plasticity of the epoxy composite can be explained if we consider TEG as a solid phase hardener^25^. In this case, a smaller number (compared to the volume of the composition) of crosslinks is formed in the border area of TEG and the epoxy composition, and, therefore, this area will have greater mobility.

When assessing the effect of the modifying additive on network polymers, it is necessary to take into account that the curing process takes place in the presence of a developed surface of the solid material (TEG), which can influence the kinetic characteristics of the polymerization reaction during curing, as well as the formation of the phase structure of the material. The role of the adsorption interaction of the components of the oligomeric composition with the solid surface of the TEG is also great^24^.

**Figure 8:**
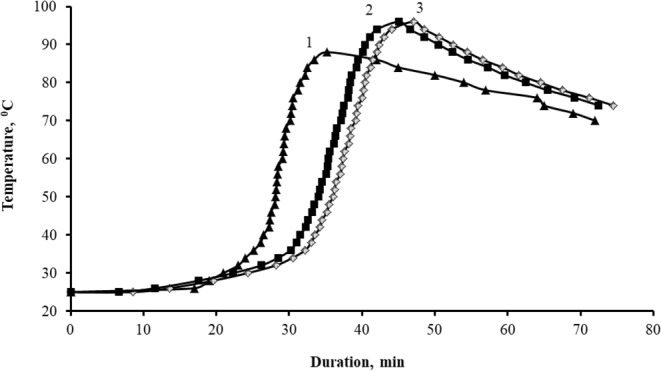
Kinetic curves of the curing process of compositions, parts by mass: 1 – 100ED-20 + 40ORPP + 15PEPA; 2 – 100ED-20 + 40ORPP + 0,05TEG + 15PEPA; 3 – 100ED-20 + 40ORPP + 1,0TEG + 15PEPA.

The study of the curing kinetics of epoxy compositions, Fig. 8, showed the inhibitory effect of TEG on the processes of the structure formation of the epoxy composite, which becomes evident in an increase in the duration of gelation processes from 25 to 31–33 minutes and curing from 35 to 45–47 minutes, Table 1.

**Table 1:** Values of the curing process of epoxy composites.

Composition, parts by mass, cured by 15 parts by mass of PEPA	τ_гел_, min	τ_отв_, min	T_max_, °С
100ED-20 + 40ORPP	25	35	88
100ED-20 + 40ORPP + 0,05TEG	31	45	96
100ED-20 + 40ORPP + 1,0TEG	33	47	95

Note: τ_gel_ is the duration of gelation process, τ_cur_ is the duration of curing, T_max_ is the maximum temperature of the self-heating of the sample during curing.

**Figure 9:**
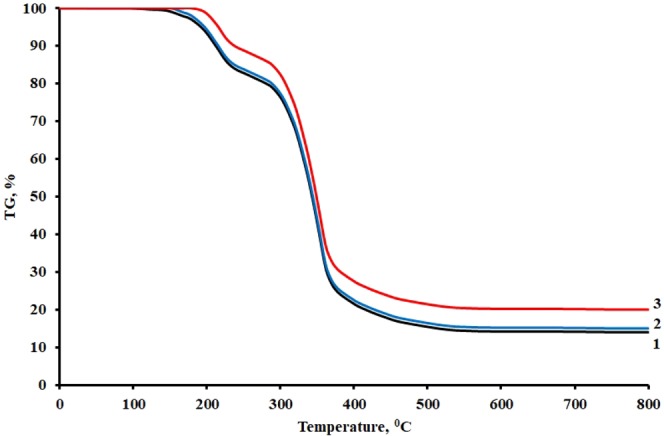
Data of thermogravimetric analysis of samples: 1 – 100ED-20 + 40ORPP + 15PEPA; 2 – 100ED-20 + 40ORPP + 0,05TEG + 15 PEPA; 3 – 100ED-20 + 40ORPP + 1,0TEG + 15 PEPA.

Figure 9 shows the data of thermogravimetric analysis of the unfilled composite and composites with the TEG additive. Epoxy composites containing TEG are characterized by better thermal stability in the temperature range of 100–600 °C compared to the unfilled composites, Table 2.

**Table 2:** Data of thermogravimetric analysis of epoxy composites.

Composition, parts by mass, cured by 15 parts by mass of PEPA	Weight loss, % at pyrolysis temperatures, °С										
	100	150	200	250	300	350	400	450	500	550	600
100ED-20 + 40ORPP	0	1,0	6,0	17,8	24,5	59,5	79,6	83,4	85,4	85,6	85,8
100ED-20 + 40ORPP + 0,05TEG	0	0	5,1	16,9	23,7	57,5	78,4	82,4	84,1	84,5	86,6
100ED-20 + 40ORPP + 1,0TEG	0	0	1,2	12,0	18,6	53,2	73,4	77,8	79,9	79,5	79,7

The addition of small amounts of TEG to an epoxy composite provides an increase in heat resistance according to Vicat from 132 to 165–182 °C, Table 3. Besides, the addition of TEG to the composition of the epoxy composite provides its increased fire resistance, which becomes evident in the reduction of mass loss during ignition in the air from 4.7 to 2.8%, and an increase in the flammability index - oxygen index from 28 to 33% by volume. The developed compounds, modified by TEG, do not support combustion in the air and belong to the class of flame-resistant materials, Table 3.

**Table 3:** Physico-chemical properties of epoxy composites.

Composition, parts by mass, cured by 15 parts by mass of PEPA	Т_V_, °С	Δm, %	OI, vol. %
100ЭД−20 + 40ORPP	132	4,7	28,0
100ЭД−20 + 40ORPP + 0,05TEG	165	4,1	28,5
100ЭД−20 + 40ORPP + 0,5TEG	171	3,6	31,0
100ЭД−20 + 40ORPP + 1,0TEG	182	2,8	33,0

Note: T_V_ – Vicat heat resistance; Δm – is the mass loss during ignition in the air; OI – oxygen index.

Thermal conductivity of compounds used in electrical and electronic equipment is an important characteristic. In most cases, epoxy resins have a relatively low thermal conductivity of ~0.1 W/m · K. Consequently, if there is local heating, epoxy materials work as thermal insulation, which requires the use of components with higher heat resistance or the use of special heat sinks to dissipate heat, otherwise it can lead to overheating and thermal decomposition of the composite^16^.

**Figure 10:**
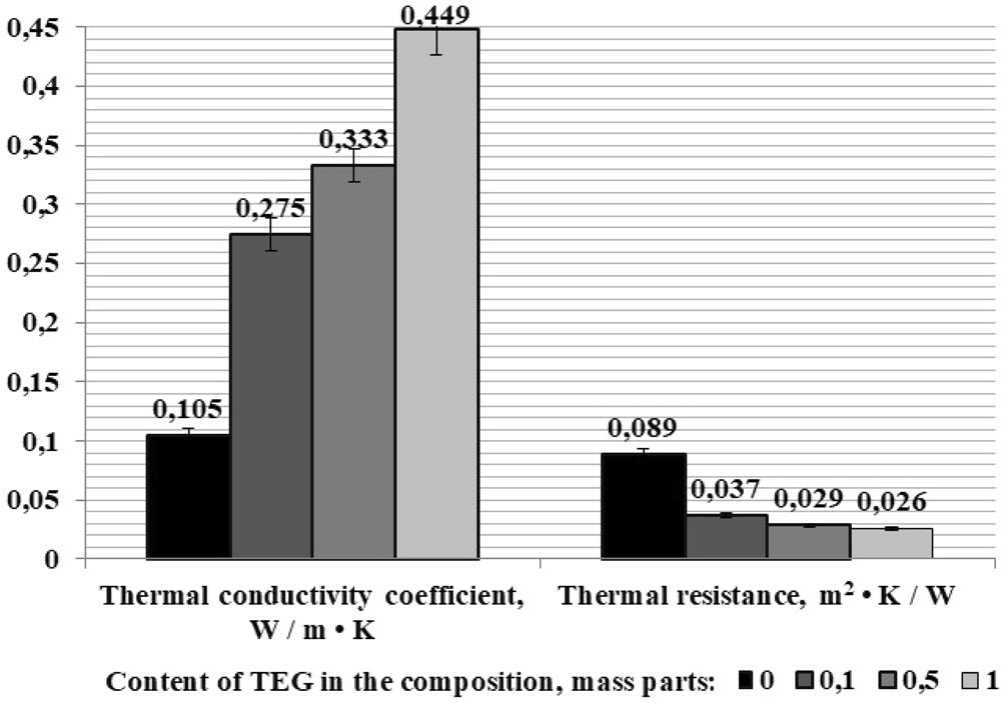
The effect of TEG on the thermal conductivity and trermal resistance of epoxy compositions.

The addition of even small amounts of TEG to the composition of the epoxy composite increases the coefficient of thermal conductivity by 2.6–4.2 times, while thermal resistance decreases, Fig. 10.

## Conclusion

As a result of the conducted studies, the possibility of directional control of the operational properties of epoxy composites with the introduction of small TEG additives into the composition, ensuring the creation of high performance epoxy composites, has been proved.

The rational content of TEG was selected as a structuring additive in the composition of the epoxy composite (0.05 parts by mass), which provides an increase in the studied complex of physical and mechanical properties. The influence of thermally expanded graphite on the process of structure formation of an epoxy composite has been established. The addition of thermally expanded graphite increases thermal, fire and heat resistance as well as the coefficient of heat-conducting epoxy composite. The developed compounds, modified by TEG, do not support combustion in the air and belong to the class of flame-resistant materials.

## Supplementary information

Supplementary Fig. S1 and S2
