# Supplementary material for: Reinforcement of Epoxy Composites with Graphite-Graphene Structures
Source: Sci Rep. 2019 Nov 7;9:16246. doi: 10.1038/s41598-019-52751-z (PMC6838145; doi:10.1038/s41598-019-52751-z)
Supplement: Supplementary file 1 — Supplementary Fig. S1 and S2 [file 41598_2019_52751_MOESM1_ESM.doc]

**REINFORCEMENT OF EPOXY COMPOSITES WITH GRAPHITE-GRAPHENE STRUCTURES**

A.S. Mostovoy*, A.V. Yakovlev**

[Yuri Gagarin State Technical University of Saratov](https://www.scopus.com/affil/profile.uri?id=60013984&origin=AuthorResultsList), 410054, Saratov, Polytechnichskaya St., 77

E-mail:* - [Mostovoy19@rambler.ru](mailto:Mostovoy19@rambler.ru); ** - [aw_71@mail.ru](mailto:aw_71@mail.ru)


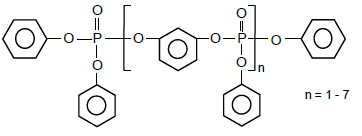


Supplementary Fig. S1. Chemical formula of oligo(resorcinophenyl phosphate) with terminal phenyl groups


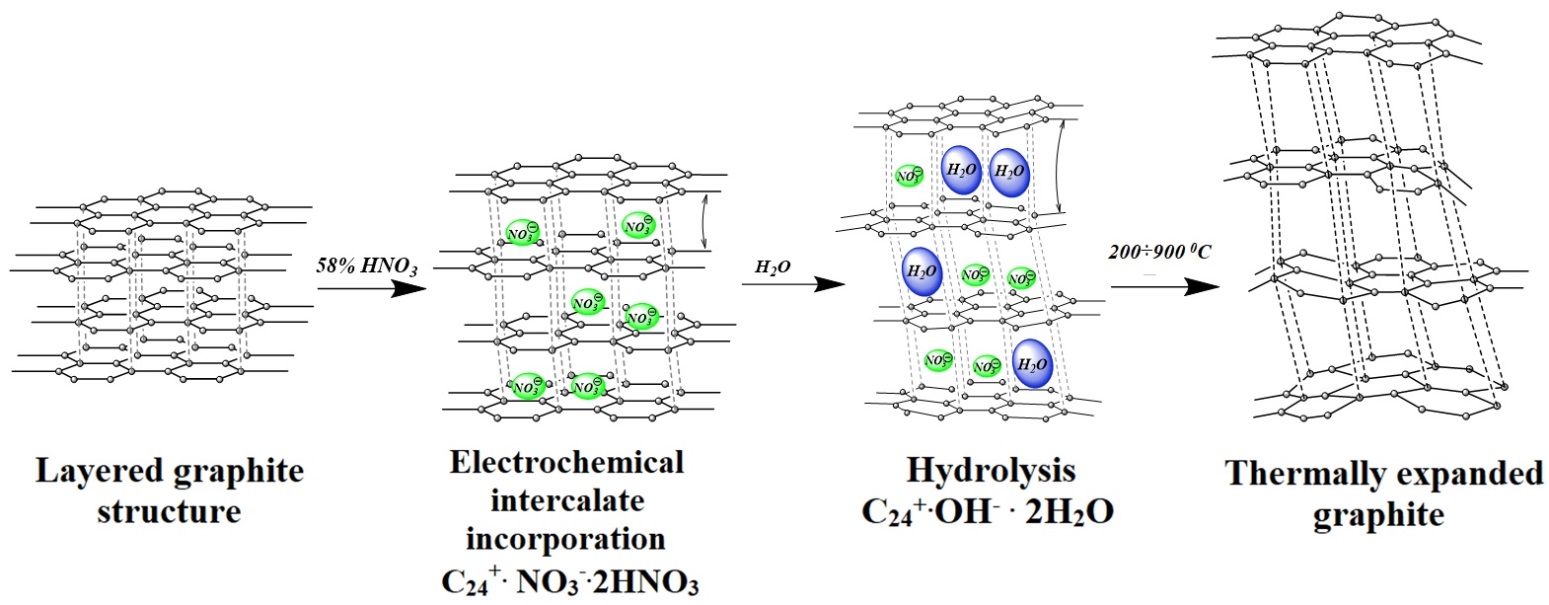


**a)**


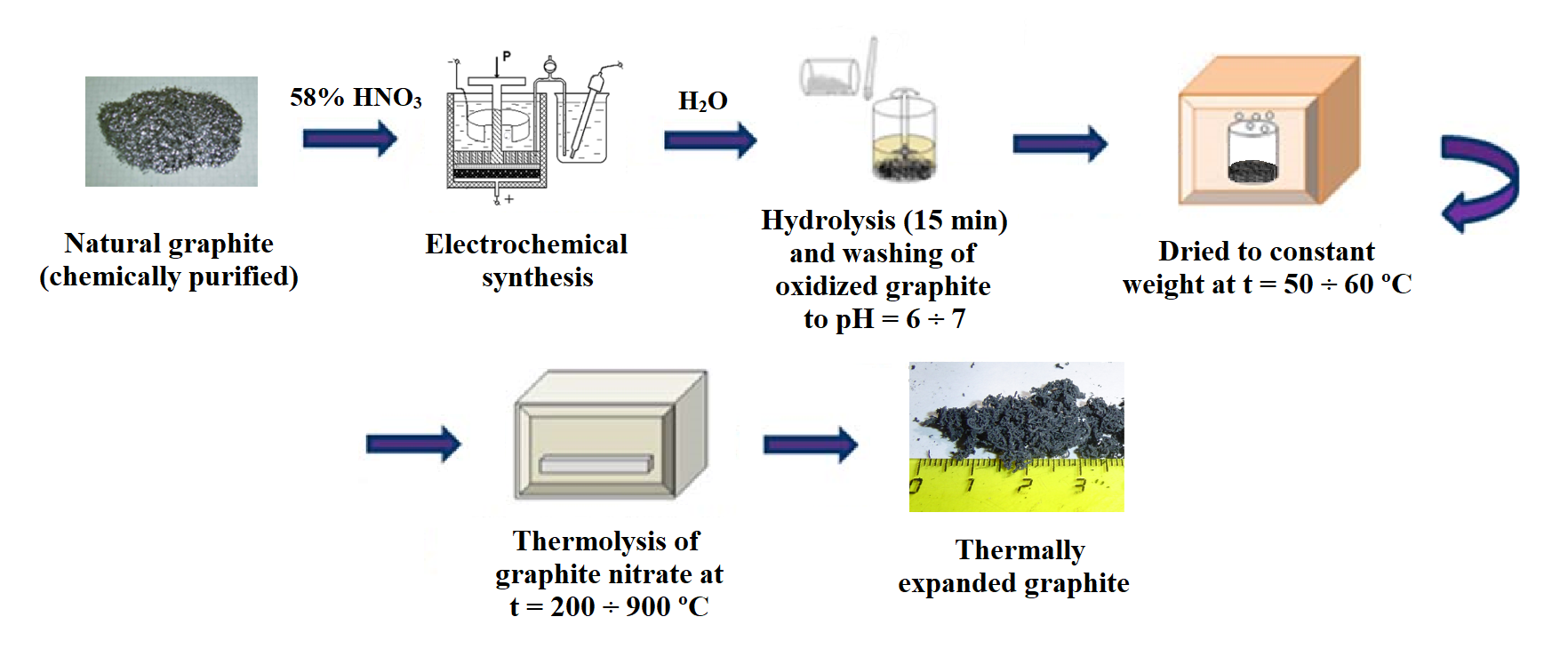


**b)**


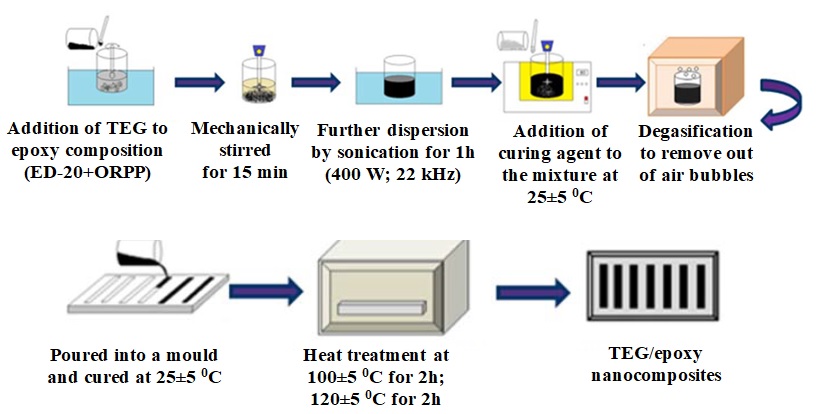


Supplementary Fig. S2. Schematic illustration for (a) the synthetic process of TEG and (b) the preparation process of TEG/epoxy composites
